# Supplementary material for: Hybrid Silica-Coated PLGA Nanoparticles for Enhanced Enzyme-Based Therapeutics
Source: Pharmaceutics. 2022 Dec 31;15(1):143. doi: 10.3390/pharmaceutics15010143 (PMC9866096; doi:10.3390/pharmaceutics15010143)
Supplement: Supplementary file 1 [file pharmaceutics-15-00143-s001.zip › pharmaceutics-2066266-supplementary.pdf]

pharmaceutics

Supplementary Materials: Hybrid Silica-Coated PLGA Nanoparticles for Enhanced Enzyme-Based Therapeutics

Kyle T. Gustafson, Negin Mokhtari, Elise C. Manalo, Jose Montoya Mira, Austin Gower, Ya-San Yeh, Mukanth Vaidyanathan, Sadik C. Esener and Jared M. Fischer

This section contains one supporting figure. Figure S1 shows fluorescently-labeled BSA encapsulated in PLGA nanoparticles as the synthesis product of the water-oil-water (W/O/W) double emulsion outlined in the main text.

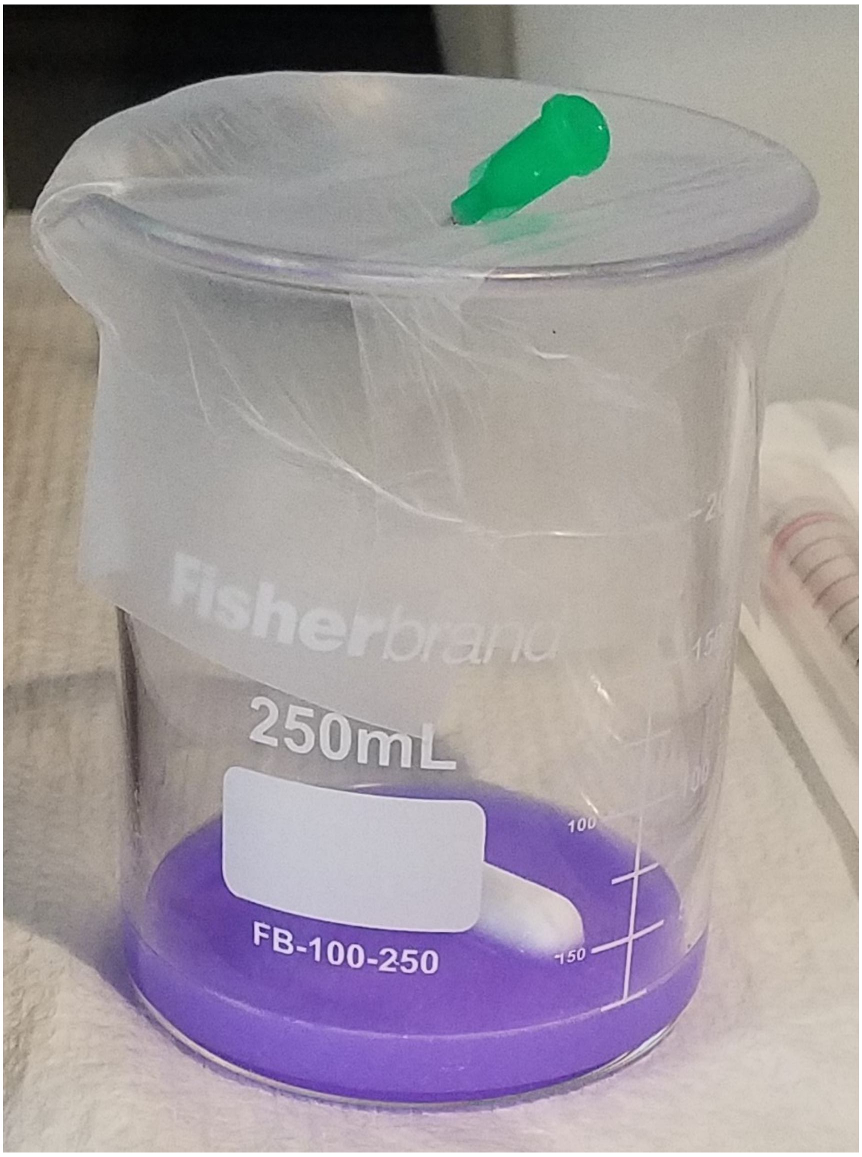

Figure S1. Synthesis product of W/O/W double emulsion: Fluorescently-labeled BSA was encapsulated in PLGA nanoparticles (NPs) after a water-oil-water (W/O/W) double emulsion synthesis. This image depicts the emulsion product (purple) immediately after emulsification, prior to overnight evaporation of the organic solvent (O) through the vented Parafilm cover and subsequent silica coating.

Disclaimer/Publisher's Note: The statements, opinions and data contained in all publications are solely those of the individual author(s) and contributor(s) and not of MDPI and/or the editor(s). MDPI and/or the editor(s) disclaim responsibility for any injury to people or property resulting from any ideas, methods, instructions or products referred to in the content.
